# Perinatal Outcome in Unbooked Teenage Pregnancies in the University of Calabar Teaching Hospital, Calabar, Nigeria

**DOI:** 10.5402/2012/246983

**Published:** 2012-03-04

**Authors:** C. U. Iklaki, J. U. Inaku, J. E. Ekabua, E. I. Ekanem, A. E. Udo

**Affiliations:** ^1^Department of Obstetrics and Gynaecology, University of Calabar, PMB 1115, Calabar, Nigeria; ^2^Department of Obstetrics and Gynaecology, University of Calabar Teaching Hospital, PMB 1278, Calabar, Nigeria

## Abstract

*Background*. Teenage pregnancy being a high risk condition requires skilled attention for good outcome. *Objectives*. To determine the influence of antenatal care on perinatal outcome in teenage pregnancies in Calabar. *Materials and Methods*. A review of patient records in Calabar was conducted between 1st January, 2006 and 31st December, 2010, to determine perinatal outcome in teenage pregnancy. *Results*. Teenage pregnancy accounted for 644 (6.5%) of the total deliveries with 245 (38.0%) booked while 399 (62.0%) were unbooked. Teenage mothers contributed significantly to the proportion of women who were delivered without prior antenatal care (*χ* ^2^ = 6.360; *P* < 0.05). The mean duration of labour in booked teenagers was 10.85 ± 4.2 hours, while unbooked teenagers was 23.31 ± 3.6 hours (*t*-value = 77.1039; *P* < 0.05). There was statistically more caesarean sections among unbooked teenage pregnancies than booked (*χ* ^2^ = 36.75; *P* < 0.05). Stillbirth was statistically significant (*χ* ^2^ = 27.096; *P* < 0.05) among unbooked teenagers than booked. However, early neonatal death was not significantly different between booked and unbooked teenage pregnancies(*χ* ^2^ = 0.512; *P* < 0.05). *Conclusion*. Unbooked teenage pregnancies were significantly associated with increased operative intervention and poor perinatal outcome.

## 1. Introduction

Teenage pregnancy is defined as pregnancy after menarche to age of nineteen [1–3]. This period corresponds to a time when there is a gradual transition from childhood to adulthood with potential conflict between biological and social factors [1]. There is also a concurrent physical, psychological, emotional, and social development [1, 2]. Among many identifiable factors contributing to the high risk of teenage pregnancies are early menarche, early onset of coital activity, early marriage, overpowering effect of the partners, inadequate parental support, single-parent factors, lack of education, poor socioeconomic background and unemployment [2]. 

There is an increased sexuality among teenagers the world over [3, 4]. In a developing country like Nigeria, teenagers account for 20 percent of the total population [5, 6]. Onset of sexual activity begins soon after menarche [1, 2]. The age at menarche has been found to be decreasing all over the world [1, 2].

A study in Nigeria in 1990 found that the median age at first intercourse was 16 years, and that by the age of 20 years; 80 percent of females had experienced sexual intercourse [5, 7]. Early marriage and childbearing is still practiced in many parts of Africa including Nigeria. Thus, more than 50 percent of young women in sub-Saharan Africa give birth before the age of 20 years [8]. In northern Nigeria for, instance, where the culture and religion permits early marriage, the minimum legal age of marriage for females is nine years [5, 9].

This increased sexual activity among these teenagers usually result in unplanned and often unwanted pregnancies [1, 10, 11]. It has been shown that 57 percent of teenage girls become pregnant within one year of sexual activity [10, 11]. A number of reasons why these vulnerable teenagers are exposed to these ill-timed pregnancies have been mentioned [12, 13]. Perhaps, the most important predisposing factor to unplanned pregnancy among teenagers is the low level of utilization of modern contraception. In a study in Lagos in 1990, only 20 percent of sexually active teenagers had ever used any form of modern contraception [14]. Another study In the eastern part of Nigeria in 1993 revealed that only 5.3 percent of sexually active teenagers had ever used modern contraceptives [15]. The rate of default among these teenagers was equally very high even when contraceptive counseling had been offered [16, 17]. A prospective study in Benin, Nigeria, found a high default rate of 43 percent among unmarried sexually active teenagers some of whom had a previous induce abortion [17]. Contraceptive use among married teenagers is equally low because of family pressure for them to become pregnant soon after marriage [17].

The inevitable result of increased sexual activity without contraception among teenagers is unplanned pregnancy. Pregnancy at a very young age is a high risk that can leads to a vicious cycle of medical, physical, and social problems from which the girl and her fetus can hardly escape [1, 18, 19]. Pregnancy at this age group is therefore generally regarded as high risk pregnancy [20, 21].

Pregnancy in teenagers are often complicated by hyperemesis gravidarum, miscarriage, malaria, anaemia, preeclampsia, eclampsia, and prematurity [1, 2]. Labour and delivery may also be complicated by obstructed labour due to fetopelvic disproportion, rupture uterus, stillbirth, obstetric fistulae, prolonged labour, instrumental delivery, caesarean section, and death [1, 2].

This trend is common in some parts of Africa due to sociocultural, geographical, and financial constraints [1, 2].

Antenatal care provides an opportunity for pregnancy complications to be diagnosed early and appropriate intervention instituted. The safest mode of delivery for the mother is determined before labour or early in the course of labour as the case may be.

In developed countries of America and Europe and some parts of the middle East where pregnant teenagers receive adequate antenatal care, pregnancy and delivery complications are minimal [2, 22].

 It is therefore important to study perinatal outcome in the unbooked pregnant teenagers and then compare with their counterparts who had antenatal care and hospital delivery.

## 2. Materials and Methods

The records of our pregnant women who delivered between 2006 and 2010 at the Maternity Annex of the University of Calabar Teaching Hospital were reviewed. During the period under review (January 1, 2006 to December 31, 2010) there were 9906 deliveries with 644 (6.5%) of this accounted for by teenage mothers. The labour and delivery records of all unbooked and booked teenage mothers were analysed. The perinatal outcome of the two groups was compared. The parameters for comparison included maternal age, booking status, duration of labour, mode of delivery, and perinatal outcome of the newborn. For the purpose of this study the following operational definitions were used:

1. a booked pregnant woman is one who attends at least one antenatal clinic session by trained personnel [23],
2. an unbooked pregnant woman is one has not attended any antenatal clinic session with a trained personnel before presentation in labour.

## 3. Results

During the period of study, 9906 women were delivered at the Maternity Annex of the University of Calabar, 7192 (72.4%) of these women were booked whereas 2714 (27.4%) were unbooked.

Teenage mothers accounted for 644 (6.5%) of women who were delivered in the hospital during the period. Out of the 644 teenage mothers who were delivered during this period, 399 (62%) were unbooked while 245 (38%) had received antenatal care. Teenage mothers accounted for 399 (14.7%) of women who delivered without prior antenatal care. 

The yearly distribution (Table 1) of the teenage mothers showed that 399 were unbooked while 245 were booked. Unbooked teenagers contributed significantly to the proportion of women who delivered without prior antenatal care (*χ* ^2^ = 6.360; *P* < 0.05).

 Age distribution of the teenage pregnant girls was classified into ≤15 years (the younger teenagers) and 16–17 years, 18-19 years (the older teenagers). More than 90 percent of the mothers were older teenagers in both groups. The mean age of unbooked teenagers was 17.4 ± 2.8 while booked was 17.2 ± 2.1, and was not statistically significant (*t*-value = 1.682; *P* < 0.05).

The duration of labour among the teenage mothers showed that 90% of the unbooked teenagers had prolonged labour while only 10% of their booked counterparts had prolonged labour. The mean duration of labour among unbooked teenagers was 23.31 ± 3.6 hours while booked teenagers was 10.85 ± 4.2 hours. Duration of labour was significantly prolonged among unbooked teenagers (*t*-value = 77.1039; *P* < 0.05).

Table 2 shows mode of delivery of the teenage mothers. Spontaneous vaginal delivery was significantly higher among booked teenagers 65.4 percent than in unbooked teenager 44.6 percent (*χ* ^2^ = 12.722; *P* < 0.05). Caesarean section was significantly higher among unbooked teenage mothers 50 percent, than in booked teenagers, 19.4 percent (*χ* ^2^ = 36.75; *P* < 0.05).

Table 3 shows the perinatal outcome of the teenage pregnancies. Live birth among booked teenagers was 96.0 percent and was significantly higher than in unbooked teenager 68 percent (*χ* ^2^ = 15.145; *P* < 0.05). There was significant higher incidence of macerated stillbirth among unbooked teenage mothers (*χ* ^2^ = 71.226; *P* < 0.05). However, early neonatal death (*χ* ^2^ = 0.512; *P* < 0.05), fresh stillbirth (*χ* ^2^ = 0; *P* < 0.05) were not significant among booked and unbooked teenage mothers.

## 4. Discussion

This study has shown that the prevalence rate of teenage pregnancy in our community of 6.5 percent is relatively high. This is however in consonance with findings in other studies in sub-Saharan Africa which show a prevalence rate of between 6–12 percent [3, 24]. Ekanem et al. found a prevalence rate of 8.3 percent among teenage mothers booking for antenatal care in University Calabar Teaching Hospital [3]. The prevalence rate is higher in Saudi Arabia, Northern Nigeria and other parts of the world where the religion and culture of the people permit early marriage [2, 25, 26]. 

It was noted that out of 644 teenage mothers who were delivered during the period, as many as 399 (62 percent) had no prior antenatal care. Teenage mothers also contributed significantly, 14.7 percent to the proportion of women who were delivered in the hospital without any prior antenatal care. This is perhaps the trend in other areas like Ilorin, where most teenage mothers are single [2, 27]. On the contrary, most married teenagers and some in stable relationships have antenatal care prior to delivery [2, 28]. This is because this group of mothers usually have spousal and parental support and approval of the pregnancy [2, 9, 29]. 

There was no significant reduction in the proportion of unbooked teenagers during the period of study, 2006 to 2010.

The proportion of unbooked teenagers were in the majority for the five years under study. This apparently suggested that the teenagers were not sensitized to the need for antenatal care during the period of study. Most of the women in our community erroneously believe that pregnancy is a natural process which requires no interference [3].

Majority of the teenage mothers as older teenagers than who have been found to have better obstetric outcome [1, 24, 30]. In the study, younger teenagers aged 15 years and below accounted for only 8.0 percent of the unbooked group. Older teenagers were therefore in the majority. Younger teenagers have however been traditionally classified as those 16 years and below while older teenagers are 17 to 19 years of age. For the purpose of this study, younger teenagers were those aged 15 years and below while older teenagers were 16 years to 19 years of age.

Majority, 90.0 percent of the booked teenage mothers were delivered within 12 hours from onset of labour. On the contrary, only 10.0 percent of the unbooked teenagers were delivered within this time limit. For the purpose of this study, prolonged labour was defined as delivery of the fetus achieved more than 12 hours from onset of active phase of labour with cervical dilatation of 4 cm. It was interesting to note that 90.0 percent of unbooked teenagers had prolonged labour. Majority of those who delivered more than 24 hours from onset of labour presented with prolonged obstructed labour. Most of those who delivered between 12 and 24 hours from onset of labour were allowed some more time in labour based on reassuring fetal well being, progress in labour and continued surveillance in labour.

Half of the unbooked teenager had emergency caesarean section for various reasons whereas only 19.4 percent of booked teenage mothers required this operation.

Unbooked teenagers accounted for 44.6 percent of teenage mothers who had spontaneous vaginal delivery whereas as many as 65.4 percent of the booked teenage mothers delivered spontaneously.

Macerated stillbirth was 29.0 percent among unbooked teenage mothers apparently from prolonged obstructed labour while 3.0 percent ended in early neonatal death from prematurity or complications of prolonged obstructed labour. Generally, booked teenagers have a better perinatal outcome than their unbooked counterpart even when subjected to the same delivery conditions [12, 31, 32]. This is because adequate antenatal care influences labour and delivery favourably [31, 32]. The high incidence of operative delivery for teenage mothers is due to fetopelvic disproportion which result from incomplete pelvic growth and development [33, 34]. Teenage mothers are also said to be likely to die as a result of complications of pregnancy and delivery [35]. No mortality was however recorded in this study among the teenage mothers probably because delivery was in hospital with adequate measures put in place to forestall such catastrophe.

## 5. Conclusion

In conclusion, the prevalence rate of teenage pregnancy in our community is relatively high. Majority of these mothers deliver without any form of antenatal care. These often render them vulnerable to various risks and hazards both to the mother and fetus. This dystocia sometimes result in stillbirth or maternal mortality. Antenatal care has been found to improve the obstetric outcome in these young women. 

To reverse this trend, promotion of moral, and sex education at home, in schools, and at places of worship. Contraceptive awareness campaigns coupled with affordable and accessible family planning services for teenagers are advocated. Teenagers should also be counseled on the need for antenatal care and hospital confinement. Emergency obstetric care should be available and accessible for management of complications of teenage pregnancy. Government should empower the citizenry and encourage the education of the girl child.

There is need for a prospective study of the obstetric performance of teenage mothers in community and possible comparison of the pregnancy outcome between the younger and older teenagers.

## Figures and Tables

**Table 1:** Yearly distribution of teenage deliveries.

Year	Booked	Unbooked	Total	**χ** ^2^
2006	60	70	130	3.609*
2007	52	92	144	0.228
2008	41	80	121	0.887
2009	40	81	121	1.275
2010	52	76	128	0.361

Total	245	399	644	6.360*

*Significant.

**Table 2:** Mode of delivery of teenage mother.

Mode	Booked	Unbooked	*χ* ^2^
	No. (%)	No. %	
Spontaneous vaginal delivery	160 (65.4)	177 (44.6)	12.722*
Vacuum extraction	28 **(**11.5**)**	22** (**5.4**)**	6.843*
Forceps delivery	9 (3.6)	0** (**0**)**	14.680*
Caesarean section	48 (19.4)	200** (**50.0**)**	36.750*

Total	245 (100)	399 (100)	

*Significant.

**Table 3:** Perinatal Outcome of teenage pregnancie.

Outcome	Booked	Unbooked	*χ* ^2^
	No. (%)	No. (%)	
Live birth	235 (96)	271 (68)	15.145*
Early neonatal death	10 (4)	12 (3)	0.5124
Fresh stillbirth	0 (0)	0 (0)	0
Macerated stillbirth	0 (0)	116 (29)	71.226*
Total	245 (100)	399 (100)	

∗Significant
